# Chicago Gynæcological Society

**Published:** 1886-08

**Authors:** 

**Affiliations:** 2330 Indiana avenue


### SOCIETY REPORTS.

TRANSACTIONS OF THE CHICAGO GYNÆCOLOGICAL SOCIETY.

*LXVI. Meeting Friday, May 28, 1886.*

*I.*—LAWSON TAIT. *Abdominal Section for Pelvic Abscess.*

*II.*—NELSON. *Specimens Removed from a Case of Supernumerary Digits.*

*III.*—PARKES. *Uterine Fibroids Treated by the Fluid Extract of Ergot.* (*Inaugural Thesis.*)

*IV.*—WAXHAM. *Occlusion of the Os Uteri as an Impediment to Labour, with a Report of Two Cases.*

The PRESIDENT, DANIEL T. NELSON, M. D., in the Chair.

I.—THE SECRETARY, DR. EDWARD WARREN SAWYER, read the following letter from MR. LAWSON TAIT.

7, THE CRESCENT, BIRMINGHAM, April 14, 1886.

MY DEAR DR. NELSON:—If not too late, I should like to take part in the discussion which was entered into at the Gynæcological Society of your city upon Abdominal Section for Pelvic Abscess. My remarks, of course, are discursive and not very conclusive, because they are based upon only a very few points to which I want to draw attention.

The first is this: I object to the use of words ending in *otomy*, to mean various operations all of which are practically identical in character but different in detail and not one of which can have any exclusive or absolute identification by any particular name. Thus

Professor Christian Fenger, in the discussion, objects to the use of the word laparotomy, and he introduces another which is perfectly new to me and I hope it will never be used again : it is *oncotomy*. Dr. Fenger objects to laparotomy in a sense where I certainly have no objections, and his very objections only show how utterly absurd all these words are. There really ought not to be any such word as laparotomy in existence, because the signification of its derivatives in the use of the people who spoke the language is such that it could not by any human ingenuity be applied to any modern surgical proceeding. Now the words "abdominal section" are sufficiently English to be understood by everybody, and they are sufficiently distinctive to enable us to understand at once that when they are used the peritoneum is opened. I therefore wish through your powerful society to protest against the use of all these stupid words of Greek formation. I wish also to protest against the absurd distinctions drawn by Sänger which are quoted by Dr. Fenger on the subject of pelvic abscess.

He distinguishes six kinds of salpingitis.

1. *Septic*, the existence of which I entirely dispute as a specific ailment.

2. *Tuberculous*, which again I deny except that it has an existence as the third and contracting stage of pyosalpinx.

3. *Syphilitic*, not one particle of evidence of this have I ever seen.

4. *Actino-Mycotic*, which is an equally ridiculous subdivision, based on mere theory, not on fact.

5. *Gonorrhœal*, to which the great bulk of the cases belong.

6. *A mixed form*. Instead of this sixth, or mixed form, I would say that there are a great many cases to which we cannot attribute any actual origin, a number of cases occurring in virgins where the existence of gonorrhœa would be an impossibility, and where there was no puerperal mischief.

Dr. Fenger's paper has always seemed to me to be an illustration of the German savant evolving the descriptions of the camel out of his own consciousness. My descriptions, on the other hand, are taken from some hundreds of cases upon which I have performed operations and the history of which I know as completely as it is possible to obtain information.

In Dr. Reeves Jackson's paper there are two points to which I

want specially to draw attention, and they are not of much importance because they are chiefly questions personal to myself.

The first is a passage in which it is said "Lawson Tait, of Birmingham, and Martin, of Berlin, were the first who attempted to prevent the terrible contingencies of pelvic inflammations by attacking the disease at its original seat; Lawson Tait removed the suppurating uterine appendages, Martin operated for suppurating periuterine hæmatocele. Tait operated for a suppurating hæmatoma of the right Fallopian tube in 1878, and he removed both tubes for pyosalpinx, and an ovary for abscess in 1885. In 1885 Martin performed laparotomy in three cases of intraperitoneal hæmatoma, namely, retrouterine hæmatocele." Now accuracy of date in a matter of this kind is rather important for one's own personal reputation, and Professor Reeves Jackson has underestimated my claim for priority by at least seven years. The first operation which I performed for suppuration of the uterine appendages was done on the 11th of February, 1872, and there will be found in the last edition of my book on "Diseases of the Ovaries," twenty-two cases which I had performed up to the middle of August, 1882, without a death. Since then I have operated upon hundreds. The first case of suppurating hæmatocele which I operated upon is published in detail in the same book; it was in February, 1879, and since then I have operated upon thirty-two cases without a death, and all have been completely cured. It will thus be seen that in none of these matters have the German surgeons approached English surgery as rivals in priority. They have been mere followers in every particular, and I regret to say their following has been practiced without that recognition to which our priority gives us every just claim.

The second point is that in which I find Professor Byford speaking in terms of my own work which no words of mine can sufficiently recognize or express my appreciation of, and here certainly his words of caution are worthy of a little note. What I fear, in fact what I already feel, is that the remarkable success which I have had, and of which Professor Byford speaks in such strong terms, is really leading astray those whose opportunities have not been as my own, into the belief that the work is easy, simple, easily acquired and free from risk. It is not so, and unless those who practice it choose to follow me in the rigid precautions and immense care which

I give, not only to the mere performance of the operation, but to the surroundings of my patients and to every detail in connection with them, they will not obtain, they must not expect, the success which I have had. I have said that I fear, in fact, I already feel, that this success of mine is leading people astray, and I want to urge in the name of humanity, as well as for the sake of the art we practice, that there should be less of the indiscriminate rushing into this kind of work which has been already deplored on both sides of the Atlantic. I am, etc.,

LAWSON TAIT.

DISCUSSION.

PROFESSOR A. REEVES JACKSON said: We ought, I am sure, to feel honored by having among us in spirit, if not in person, so eminent a man as the writer of this letter. Lawson Tait is in some respects the greatest living surgeon, a Gamaliel at whose feet we all find ourselves sitting; and, withal, a man so observant that not a single gynæcological sparrow falls in any part of the world unnoticed by him. I must plead "not guilty" to the charge—made against me by Mr. Tait—of inaccuracy regarding the date of his first laparotomy for pelvic abscess,—the remarks upon this point having been made by another, and not by me. As stated in the letter, his first operation of this nature was done February 11, 1872, at Birmingham, on a patient of Mr. Hallwright. I am sure that Mr. Tait will not for a moment suppose that any of us would willingly do injustice to one whom we all esteem so highly, and from whom many of us have been recipients of acts of kindness and courtesy.

In regard to the justice of Mr. Tait's criticism on the prevalent use of the words ending in "otomy" I do not feel like being an arbiter. Technical words are frequently necessary, and yet, as a general rule, I think it preferable to use simple language. The ordinary English words are commonly sufficient to answer all the purposes of language. Besides, large and unusual words are sometimes embarrassing. When, some weeks ago, Professor Fenger charged me with performing an "oncotomy" I was afraid that I had done something very dreadful, and the worse because I did it without knowing it. I felt very much as the fisherwoman did when Daniel O'Connell in response to her volley of ordinary, undecorated profanity called her a parallelogram. The fisherwoman did not know what to say, and I could not reply; we both had evidently lost the thread of the discussion. I am glad that Mr. Tait speaks so strongly in regard to the tendency now so frequently indulged in, to perform laparotomies, and that he is willing to correct to some extent by his words the mischief that has been done by his powerful and successful work. It seems that when some persons visit Mr. Tait and witness his success and simple but effective methods, they come back thinking life is a blank unless they can own and manage an abdominal hospital and spend the remainder of their days in the cheerful occupation of removing uteri and ovaries.

PROFESSOR CHRISTIAN FENGER said:

The letter which Mr. Lawson Tait wrote to Dr. Nelson relates in a number of points to my paper on "Laparotomy for Periuterine Abscess" as well as to some remarks which I made before the SOCIETY in a previous discussion. I must, therefore, beg the FELLOWS of the SOCIETY to bear with me if I take up a little of their time in answering Mr. Tait's letter.

Discussing Professor A. Reeves Jackson's paper, I objected to calling the operation in question a laparotomy. According to the Professor's description of the case, he had opened an abscess which was adherent to the anterior abdominal wall. He had consequently simply performed an oncotomy, an operation which, notwithstanding the division of the abdominal wall, does not differ materially from opening a deep-seated abscess in any other region of the body, as *ex. gr.* in an extremity.

Whether opening the abdominal or peritoneal cavity be termed laparotomy or abdominal section or *Bauchschnitt*, is of course a matter of indifference, provided only that the meaning of the word be agreed upon. There is but one way of getting at the signification of a medical term, and that is by learning in what sense the term is employed in the medical literature of the different nations.

I must again maintain that laparotomy is not merely section of the abdominal parietes, but that the word implies opening of the general peritoneal cavity with a view to performing some operation within that cavity. (See *Linhardt's Operationslehre*. Wien. 1862, p. 705, and *Eulenburg*, *Realencyclopædie*, Bd. II, p. 37.) French authors occasionally use the word gastrotomy instead of laparotomy. Recently the operation has been called peritonotomy, which on account of its correctness should perhaps be preferred to the other terms.

It is of importance to distinguish between a laparotomy and the evacuation of a limited abscess by simply incising the abdominal wall. The two operations differ widely as to their consequent dangers. Where the general peritoneal cavity is opened, a well-known series of precautionary measures is required before and during the operation, in order to protect the patient from general septic peritonitis.

Where an incision through the abdominal parietes leads directly into a limited abscess-cavity, the precautionary measures essential to laparotomy may be dispensed with; the general peritoneal cavity is not opened and there is no fear of general peritonitis. In the latter operation the peritoneum is not seen or not taken notice of, the incision-wound is left open and the limited cavity washed out and drained.

In every country medical authors hold these two operations apart. Opening a perityphlitic abscess, an adherent hepatic abscess or a parametritic abscess above Poupart's ligament is never spoken of as a laparotomy. Whenever we hear or read of a man having performed a series of laparotomies we naturally suppose that he is experienced in performing intraperitoneal operations. Now, many a surgeon has opened a large number of intra-abdominal abscesses and has never seen the peritoneal cavity.

It is evidently important properly to limit the meaning of the term laparotomy; else we may misunderstand the description of a given case, and the statistics of laparotomies will necessarily be rendered valueless.

The next point Mr. Lawson Tait remarks upon is the question of priority of operating for pelvic hæmatocele.

By the mistake of Mr. Tait, part of what I said in the discussion of Professor Jackson's paper is ascribed to the paper itself. I am thus quoted in his letter:

"Lawson Tait, of Birmingham, and Martin, of Berlin, were the first who attempted to prevent the terrible consequences of pelvic inflammations by attacking the disease at its original seat. Lawson Tait removed the suppurating uterine appendices; Martin operated for suppurating periuterine hæmatocele."

In the discussion I stated the dates at which Tait and Martin performed their respective operations. By a typographical error the dates appeared wrong in some of the copies of the transactions of the SOCIETY. 1885 instead of 1872 was given as the date of Tait's first laparotomy for abscess of the ovary, and 1885 instead of 1876 as that of Martin's first laparatomy for extraperitoneal hæmatocele.

Considering my remarks as a whole and examining my references to the literature on the subject a careful reader would not have failed to recognize in the wrongly printed dates a typographical error.

Lawson Tait performed his first laparotomy for extraperitoneal hæmatocele in February, 1879. (See LAWSON TAIT: *Diseases of the Ovaries. 4th Edition. London, 1883, p. 346, and Tait's letter.*)

Martin performed the same operation for the first time in 1877, and a second time on the 31st of January, 1879. (See A. MARTIN: *Das extraperitoneale periuterine Hæmatom. Zeitschrift fur Geburtshulfe und Gynekologie. VIII Bd., Hft. II, 1882, p. 476.*)

It will thus be seen that my statements concerning priority were correct. In the discussion of Professor Jackson's paper I quoted Sänger's classification of pelvic inflammations. In criticizing it, Mr. Lawson Tait speaks of "absurd distinctions." I wish to repeat that I regard Sänger's classification as complete and correct and in accordance with the laws governing inflammatory processes in all organs of the body.

To Mr. Lawson Tait's manner of criticizing my little paper "On Chronic Periuterine Abscess and its Treatment by Laparotomy," which appeared in the May number of the *Annals of Surgery*, 1885, there is but one answer.

Like all published articles it is open to criticism. If anybody should wish to attack it, I am ready to enter into a discussion of it, provided that tangible objections to it be brought forward.

II.—The PRESIDENT exhibited specimens removed from

### A CASE OF SUPERNUMERARY DIGITS.

He said: While I know the condition is not exceedingly rare, I thought the specimen was so beautiful as to be worthy of presentation to the SOCIETY. The specimen consists simply of two supernumerary little fingers, which I found in a beautiful, healthy baby just after it was born, attached by small pedicles, consisting simply of the skin and the vessels needed to supply them, about the middle of the first phalanx of the little finger; the pedicles were perhaps one-sixteenth of an inch in length, just long enough to ligature. They look like little beans; the finger nails are fairly developed in both.

They were very vascular. They looked, before removal, like bangles. This was the sixth pregnancy; the other children were all perfect; no other case of this condition in the family that is known. The condition is usually hereditary. In one there is a very good nail formed, upon the other there is only a slight nail. The mother is in good vigor and health. They were united to the larger little finger about the middle of the first phalanx—one was just about the middle of the phalanx, on the outer border; the other, half way between the middle line and the outer border. They both feel as if there are bones in them—two phalanges in each, the third being represented by the pedicle.

III.—PROFESSOR CHARLES T. PARKES (Rush, 1868,) read the following paper, entitled

UTERINE FIBROIDS TREATED BY THE FLUID EXTRACT OF ERGOT.

My intention is to relate to you the history of four cases of uterine tumor, and to present a few remarks suggested by them. These four cases were treated by the internal administration of Squibb's fluid extract of ergot. They all resulted in recovery by expulsion of the growth.

I found no insurmountable difficulty in giving the medicine, although when given for a prolonged period it creates nausea and disgust in some. This was counteracted, and the pain following its use controlled by combining it with morphine. It seemed to me preferable to the hypodermatic use--the latter being locally painful and often producing abscess, besides it is not followed by any better result. Two of the cases, treated by ergot, when thrown off, proved to be pure uterine fibroma—dense and hard—white and glistening when cut open—consisting of simple fibrous tissue. The other two following the action of ergot were soft myomata—pultaceous and semi-elastic—consisting mostly of connective tissue, confirming the diagnosis made. All four of these were evidently submucous tumors, or so slightly interstitial as to be practically covered only by mucous membrane.

CASE I.—Mrs. S., American, 43 years old, widow, three children, no miscarriages, menstruated first when 16 years old. Never had any noticeable trouble with menstruation until three years previous to my first examination; during these years she had suffered with irregular profuse hæmorrhages which were now continuous, accompanied with exacerbations on the slightest exertion. My first examination was made February 20, 1876. As my memory brings this patient before me she presented the most perfect example of transparent flesh that I had ever seen. A large, finely formed woman, her flesh looked like alabaster, apparently destitute of blood. The legs were œdematous, the heart beat feeble and rapid, and the slightest exertion was followed by extreme palpitation and the most fearful feelings of suffocation. Her answer as to what she had done for her trouble was that she had taken "quarts of medicine." Vaginal examination revealed an enlarged uterus and patulous os, from which blood was rather freely oozing. The sound entered the uterus about five inches, the handle being deviated forward and to the left side. A diagnosis of submucous uterine fibroid was made.

The treatment adopted was the administration of strychnia and iron, together with wine and good diet for the general condition, and one-half drachm doses of Squibb's fluid extract of ergot every six hours, to either expel or kill the growth. Locally, to stay the hæmorrhage, a small tampon of pulverized alum was applied to the *os uteri* and held in position by ordinary cotton tampons.

The first forty-eight hours' use of the ergot produced quite severe uterine pains, so acute that the patient, in her weakened condition, said they were unbearable. At this visit Professor T. D. Fitch was with me in consultation. The tampon of alum was removed, and Dr. Fitch's examination confirmed the diagnosis made, and advised the continuation of the treatment. As the bleeding had been entirely controlled by the tampon, it was left out. The ergot was continued as before, and a sufficient dosage of morphine ordered, to make the pains bearable if they persisted. No further use of the tampon was required, the uterine contractions never ceased while the ergot was administered. On the sixth day of its use a foul-smelling serous discharge came on, *per vaginam*, accompanied with slight general chilliness and a temperature of 102° F. The patient was assured that the tumor was surely coming away, and encouraged to bear "yet awhile" with her great suffering. On the eighth day the tumor was found in the vagina and removed. It was about the size of a duck's egg and very hard to the touch. After a short period of mild septic trouble the patient passed through a quick convalescence and rapidly recovered. Seen a few months ago she says she has never had any illness since getting rid of this growth, and certainly looks well.

CASE II.—Mrs. P., German, married, five children, no miscarriages, menstruation began at 14, now 37 years old. Seen by myself first in March, 1881. The patient is a robust, hearty woman. Never had any trouble until six months after the birth of last child, about one year ago, when she began to flow too freely and too often—as often as every week or occasionally twice a week. The blood was in large quantity and bright red in color.

The examination revealed an enlarged uterus—it could be felt above the pubis during bimanual examination. The sound entered easily for five inches, the handle deviating forward and to the left. Its use was accompanied and followed by very free bleeding.

*Diagnosis.*—Submucous uterine fibroid on anterior wall. *Treatment.*—locally the alum tampon was used as in the previous case. Squibb's fluid extract of ergot in half drachm doses every six hours. The patient was ordered to remain in bed. On the succeeding day all the tampon, except the alum, was removed. No hæmorrhage; slight pains complained of. On the second day the pains were very severe and morphine was given to control them. The alum tampon was removed and a carbolized hot water injection ordered three times a day. On the third day pains still severe and a foul-smelling vaginal discharge commencing.

This condition persisted until during the night of the eighth day, when I was summoned to the patient on account of the unusual severity of her sufferings, the messenger, her husband, saying it was just as if she were having a baby. On my arrival the pains had quite ceased. Examination showed the loss of considerable blood, and the vagina was found filled with a large fleshy mass, horribly offensive. The finger could be passed beyond it, and the largely opened cervix recognized. It was seized with a Vulsellum forceps, twisted a few times upon itself, and then delivered. The mass was as large as a closed fist, dark colored and ragged all over its surface and very foul smelling. The patient rapidly regained her usual health, and is well today.

CASE III.—Mrs. E., 33 years old, married, three children living, one miscarriage, menstruation commenced when 14 years of age. Was first called to visit her January 2, 1885, for severe uterine hæmorrhage. She then informed me that she never had any trouble with menstruation until about two years previous.

Shortly thereafter she was operated upon for laceration of the cervix, without much relief to her trouble, since she had gradually grown worse, so that she was not free from bleeding ten days in the month.

One year previous to my seeing her, the uterus was freely curetted and fuming nitric acid applied to the cavity as a relief for the bleeding. The procedure failed in any good result.

At this visit the bleeding was extreme in degree. Examination revealed the pelvis largely filled with a smooth, doughy mass. After considerable searching the *os uteri* was found high up above and close to the pubis. It could only be found by crowding the finger between the bone and the growth. The growth was exquisitely tender to the touch or any manipulation. Bimanual palpation discovered an uncertain mass above the pubis. The vagina was tamponed temporarily, and morphine administered hypodermatically. The diagnosis was reserved. In my mind it rested between hæmatocele and soft myoma. The tumor was compressible, at least its elements seemed to give way to the pressure of the finger; it was semi-elastic and painful under manipulation, filled the entire posterior half of the pelvis, and the os was carried well upward and forward. It might be, and probably was, a myoma of the posterior uterine wall retroverted.

(A few weeks ago I had the satisfaction of seeing the *fac simile* of this case, so far as the character of displacement and the position of the tumor was concerned, in a patient under the care of Dr. Merriman, although the tumor in Dr. Merriman's case was much harder and more resistant.

Dr. Merriman, with skill and apparently with ease, lifted the tumor out of the pelvis into the general abdominal cavity, after placing the woman in the knee-elbow position—a change bringing much comfort to the patient. I learn that under the use of ergot the growth is already diminishing in size.)

The patient was put upon fluid extract of ergot in one-half drachm doses three times a day. The next menstrual period showed no change other than a diminished loss of blood. In June the flooding was quite free and accompanied with considerable pain. In July everything was as bad as possible, with so much pain that the ergot was discontinued. Repeated examination now narrowed the diagnosis down to soft myoma. The removal of the uterine appendages was suggested, in the hope that this procedure would anticipate the menopause, stop the bleeding and lead to the gradual atrophy of the growth. In September, consultation was solicited with Professor W. H. Byford, when the patient was etherized and carefully examined. The sound, introduced with great difficulty, owing to the displaced position of the os, passed in over five inches, positively demonstrating the nature of the growth, its consistency showing it to be the soft variety of myoma.

As the patient could not be said to be in absolute danger of her life, the operation was refused by her friends, although the sufferer was willing enough to have it done.

The previous treatment was endorsed by Professor Byford, and its continuance advised.

The fluid extract of ergot was resumed and rendered bearable by morphine.

The October illness was accompanied by slight hæmorrhage, but excessive pain. These uterine contractions continued on after the menstruation ceased, until, during the last week of October, they became labour-like in character. Examination now revealed that the uterus had righted itself, the os was becoming patulous and its edges thinned out; through its opening the projecting tumor could be felt. On the second day of November, pieces of the broken-down mass, horribly offensive, could be seized with the forceps, pulled out of the uterus and cut away.

Chilly sensations began to be felt by the patient, sweatings came on, the temperature ran up to 101° F., and a mild scepticæmia was established. During the following week the pains never ceased. Quinia was administered freely. The vagina and uterus were irrigated with hot carbolized injections, and the mass removed as fast as any of it could be reached, and at the week's end the last remnant was gotten away. The patient was very much reduced physically, but rapidly convalesced, and is now perfectly well, with her menstruation normally re-established. Fully a quart of soft pultaceous pieces of the growth were removed.

CASE IV.—Mrs. L., German, 34 years old, one child, was seen first November, 1885. She had been suffering with increased menstrual flow for a year. She came to me to be treated for an external, painful, labial swelling. It proved to be a vulvo-vaginal abscess. It was opened freely and gave no further trouble. A uterine tumor was noticed and examined. It was found to be of considerable size,—could be detected above the pubis.

She was put upon one-half drachm doses of fluid extract of ergot every six hours.

This she continued for six months steadily, with varying conditions of pain and hæmorrhage, until in April, 1886, the hæmorrhage ceased, pain became very severe, and a shreddy, foul-smelling discharge manifested itself. She was removed to the St. Joseph's Hospital, and after ten days of antiseptic washings and removal of masses of broken-down tissue, the mass was entirely extruded. This patient had also quite a severe septicæmia, but finally recovered and is now well.

*Remarks*:—Aside from crucial demonstration, it seems reasonable to assert that these four cases were cured by means of the remedy used, and by that alone. It is well known that the most of authorities state that no positive reliance can be placed on the use of ergot. My experience surely, as here illustrated, and confirmed by other cases seen, leads me to think that this adverse judgment must be qualified, especially in the treatment of the submucous bleeding fibroids of the uterus. If the growths be partially interstitial, the less the thickness of uterine tissue between them and the mucous covering, the more certain will the remedy be curative. Of course it will be impossible to demonstrate the exact amount of uterine wall forced contractions will destroy, hence a trial of its worth is desirable in all cases not purely sub-peritoneal and pedunculated. Still I am quite convinced the severity of the hæmorrhage gives one a good reason to speak *positively* of the results to be accomplished by its use. I have not deemed it necessary to look up the history of the first use of this remedy for stimulating uterine contractions. It is sufficient for me to say that my confidence in the remedy and persistence in its use, even when failure of good results seemed certain, has followed directly as the result of the teachings and experience of an honored Fellow of this SOCIETY, Professor Wm. H. Byford, who has never lost an opportunity to urge upon the profession his belief in the specific action of the remedy, and its absolute certainty of cure in many cases. I am quite sure that Professor Byford deserves the credit of being the first to make use of ergot with the idea of destroying the vitality of the growth, and as well, causing its expulsion from the uterus.

The third case shows plainly how absolutely unnecessary any operation would have been. The removal of the appendages might have stopped the hæmorrhage, but such a perfect cure as now exists could never have followed operation, to say nothing of the harm done by unsexing the woman; still no case could present better reasons for such a procedure, none in which it would have been more justifiable from the indications present. To me it brings the lesson to make oöphorectomy the *dernier ressort* in all cases, certainly to give the remedy used at least a six months' trial without result before operation be sanctioned.

The difficulties attending the differentiation between a distended sack of fluid and the soft myomata was well illustrated by this case. The sensation communicated to the touch was scarcely distinguishable from fluctuation. It was only after repeated examinations under ether, and the use of the sound, that the diagnosis was satisfactorily settled.

The fourth case for a time seemed one in which the treatment would come to nothing. Every one became discouraged. The suffering was increased, and no advance was made, apparently. By persistence the cure was accomplished. In this case operative interference was solicited by the patient, and would have been most readily submitted to, without any urging. If I read aright the indications which authorities give, to justify the resort to removal of the uterine appendages, they were all present in this case, and more too if that were needed. Certainly the final result has proven any such interference would have been uncalled for and lamentable.

I am quite well aware that four cases cannot be considered absolutely demonstrative of any rule, still these four increase the number already published in proof of the curative action of ergot, administered thoroughly, for sub-mucous uterine growths. It is impossible for me to understand how some good authorities can still assert their disbelief in ergot; in fact, calling it the most inert and disappointing of all drugs. No possible argument can disabuse my mind of the belief that its action was positive and certain in the cases related. No law has as yet been evolved fixing even by approximation the period of time required for the effects of the medicine to show themselves. The idiosyncrasies of the patient, the thickness of the uterine envelope, the distance from the mucous membrane, the purity of the drug, and many other conditions, render it scarcely possible that any such law can ever be laid down. The trial should be made patiently and persistently, just so long as the patient's condition will warrant its continuance, and a complete expulsion of the growth, followed by rapid recovery in many cases, will be the reward.

IV.—PROFESSOR F. E. WAXHAM read the following paper, entitled

OCCLUSION OF THE OS UTERI AS AN IMPEDIMENT TO LABOUR, WITH A REPORT OF TWO CASES.

Having met with occlusion of the os but once in several hundred cases of labour, and knowing of a number of physicians of extensive practice who have never seen this condition present at the time of confinement, I am convinced that it must be of rare occurrence, and the history of two cases may not be uninteresting.

Mrs. S., primipara, twenty-nine years old, German, fell in labour about 9 P. M., February 21, 1885. The membranes ruptured soon after the commencement of labour and the amniotic fluid gradually drained away.

The patient was seen between 3 and 4 A. M., at which time the pains had become very severe and frequent. Upon examination the head was found low down in the inferior strait, almost presenting at the vulva, and covered apparently by a thin membrane through which the advancing head threatened to burst with every pain. Upon the most careful digital examination, no os could be discovered nor the slightest indication of one. Professor Nelson was summoned and promptly responded. His more experienced finger detected a very slight dimple in the center of the presenting tissues. By keeping the finger upon this slightly thickened tissue, he discovered that it became very much thinner with every pain, while as the pain subsided the tissues assumed a very slightly umbilicated appearance. By firm and continued pressure upon this suspicious spot an opening was at length effected and the os gradually dilated. As the labour proceeded slowly, and fearing the result to the child of so long a delay of the head in the pelvis, and the os being fully dilated, the forceps were applied. The child was delivered without injury to the mother, but it was asphyxiated and required considerable effort in resuscitation. Professor Nelson stated that this was the second case that had ever come under his observation, and kindly gave me the history of the following one. He was called to attend a lady in her first confinement, a Swede, twenty-three years old, and married about one year. Making a hasty examination, he found a well-formed cervix but did not detect the os. On returning a few hours later, the head had descended to the inferior strait and was indeed presenting at the vulva and covered by the cervix, which had become so thin as to resemble the membranes. The membranes had already ruptured and the amniotic fluid had gradually escaped. There was no appearance whatever of the os. It could not be detected with the finger, and the head seemed about to burst through the uterine tissue. The patient was placed before a window, the labia separated and careful search made for the os. Only after a most careful search was it found. It was patulous only to the extent of admitting the very finest surgeon's probe. After this had been introduced and worked about, a second probe was passed, and by separating them the os was gradually and sufficiently dilated to allow the finger to enter. The os was then rapidly dilated and labour progressed normally.

I find the literature on this subject quite meager, many of our writers on obstetrics omitting the subject entirely, while others refer to it very briefly.

Schroeder alludes to it in the following terms:

"As complete atresia of the os prevents conception, it follows that an "occlusion of the os, observed in labour, must have taken place during "pregnancy.

"Very frequently there is a superficial and easily separable agglutination "of the external os. It is due to an inflammatory process of the lips of the "os from a previous blennorrhœa. During labour the advancing head is seen "to push the lower uterine segment forward to the outlet, and to thin it more "and more. This thinning may be so great that the head appears to be "covered only by the membranes. By an accurate examination the os feels "like a small and soft dimple directed greatly backwards. If during a pain "the finger or uterine sound be forcibly pressed against the dimple, the "agglutination of the os will suddenly give way. The os itself now very "rapidly dilates and labour proceeds without impediment. Often the pains "themselves succeed in breaking down the adhesions of the os.

"It very rarely happens that the os only partially dilates after the agglu-"tination has been torn through and remains rigid so as later to require "incisions. There is very seldom so firm an adhesion between the maternal "and fœtal membranes in the immediate vicinity of the internal os that the "lower uterine segment cannot retract over the ovum. Separation by the "finger or rupture of the membranes renders possible the dilation of the os."

Schroeder also refers to the fact that a firm cicatricial band may occasionally occlude the os, resulting from inflammation of the cervix or cauterization:

When these firm adhesive bands prevent dilation of the os there is danger of rupture of the vault of the vagina unless incisions are made and assistance given. The cicatricial closure of the os, is frequently incomplete; more or less fine openings remaining pervious, rendering conception difficult but still possible, is believed by Schroeder to frequently result from ulcerative inflammation during the lying-in state.

Leishman, in discussing this subject, remarks that

"There are some cases in which there seems to be actual occlusion of "the os. Impregnation in the case of an absolutely occluded os is as impos-"sible as that the normal function of menstruation should be carried on, "and therefore we must assume, in such cases, that the closure must have "taken place subsequently to the entrance of the seminal fluid. It is of "course possible that the os may remain open to a very limited extent, and "yet the state of the tissues renders distension impossible, so as practically "to constitute an impediment as insurmountable as actual occlusion would "be."

Playfair gives the following brief intention of this condition :

"Agglutination of the margins of the os uteri is occasionally met with and "must of course have occurred after conception. It is generally the result "of some inflammatory affection of the cervix during the early months of "pregnancy. Usually it is not associated with any rigidity or hardness, but "the entire cervix is stretched over the presenting part and forms a smooth "covering in which the os exists only as a small dimple and may be very "difficult to detect at all. Occlusion of the os from inflammatory changes "sometimes so alters the cervix that no sign of the original opening can be "discovered."

All our authorities agree that the occulsion of the os is the result of inflammatory change occurring subsequent to impregnation. It is a noteworthy fact that in both these cases the membranes ruptured and the amniotic fluid escaped in the very early stages of labour, showing that the membranes were adherent to the uterine tissue about the internal os. As the internal os dilated, rupture of the adhesions and of the membranes necessarily followed.

The discussion of the papers read by Professor Parkes and Professor Waxham was, on motion, deferred until the June meeting.

Mr. Lawson Tait, of Birmingham, and Professor T. Gaillard Thomas, of New York, were elected Honorary Fellows of the Society.

Professor E. C. Dudley proposed for honorary fellowship Protheroe Smith, M. D., M. R. C. P., of London.

W. W. JAGGARD, M. D.,

